# Supplementary material for: Universal and Non-Universal Features in the Random Shear Model
Source: Entropy (Basel). 2022 Sep 24;24(10):1350. doi: 10.3390/e24101350 (PMC9600650; doi:10.3390/e24101350)
Supplement: Supplementary file 1 [file entropy-24-01350-s001.zip › entropy-1878712-supplementary.pdf]

Supplemental Material - Universal and non-universal features in the random shear model

Fabio Cecconi[1] and Alessandro Taloni[1]

[1]Istituto Sistemi Complessi, Consiglio Nazionale delle Ricerche, via dei Taurini 19, 00185 Rome, Italy

Contents

1 Third moment calculations **1**

2 Fourth moment calculations **2**

1 Third moment calculations

By inverting the Laplace transform expression (29) (main text), it is immediate to see how the main contribution arises from the first addendum. This is because the summation over two indices, n_1 and n_2, surmounts the second summation performed over a limited set of n_1. Thus, inverting the first term in time yields

$$\langle\langle[x(t)-x(0)]^3\rangle_w\rangle_0 \simeq \frac{3!}{2^3}\sum_{\substack{n_1,n_2=-M\\ n_1\neq -n_2/2}}^{M} U_{k_{n_1}}U_{k_{n_2}}U_{k_{-n_2-n_1}}\times$$

$$\left\{\frac{t}{k_{n_1}^2(k_{n_1}+k_{n_2})^2D^2}+\frac{e^{-k_{n_1}^2Dt}-1}{k_{n_1}^4(k_{n_2}^2+2k_{n_1}k_{n_2})D^3}-\frac{e^{-(k_{n_1}+k_{n_2})^2Dt}-1}{(k_{n_1}+k_{n_2})^4(k_{n_2}^2+2k_{n_1}k_{n_2})D^3}\right\} \tag{S1}$$

The long time behaviour gives $\langle\langle[x(t)-x(0)]^3\rangle_w\rangle_0 \sim L^2t$. For short times $t \ll \frac{L^2}{M^2D}$, the third moment behavior can be assessed by expanding the exponential functions in (S1) to the third order, yielding $\langle\langle[x(t)-x(0)]^3\rangle_w\rangle_0 \sim L^2t^3$. For intermediate times $\frac{L^2}{M^2D} \ll t \ll \frac{L^2}{D}$ a direct calculation shows that the leading contribution is attributable to

$$\langle\langle[x(t)-x(0)]^3\rangle_w\rangle_0 \simeq \frac{3!}{2^3}\sum_{\substack{n_1,n_2=-M\\ n_1\neq -n_2/2}}^{M} \frac{|k_{n_1}|^{\gamma/2}|k_{n_2}|^{\gamma/2}|k_{n_2+n_1}|^{\gamma/2}}{k_{n_1}^4(k_{n_2}^2+2k_{n_1}k_{n_2})D^3}\left(e^{-k_{n_1}^2Dt}-1\right). \tag{S2}$$

Tracing the analysis accomplished in Ref.[1], we assume large channel widths $L \gg 1$, so the summations over k_{n_1} and k_{n_2} can be replaced by a double integral over the interval $\left[\frac{2\pi}{L},\frac{2\pi M}{L}\right]$ and approximated by:

$$\langle\langle[x(t)-x(0)]^3\rangle_w\rangle_0 \simeq \frac{3L^2}{8\pi^2D^3}\int_{\frac{2\pi}{L}}^{\frac{2\pi M}{L}}dk_1\int_{\frac{2\pi}{L}}^{\frac{2\pi M}{L}}dk_2\frac{k_1^{\gamma/2-4}k_2^{\gamma/2}(k_2+k_1)^{\gamma/2}}{k_2^2+2k_1k_2}\left(e^{-k_1^2Dt}-1\right). \tag{S3}$$

Applying the change of variables $y = Dk_1^2t$ and $z = Dk_2^2t$, the time scaling expression is immediately achieved: $\langle\langle[x(t)-x(0)]^3\rangle_w\rangle_0 \sim L^2t^{2-\frac{3\gamma}{4}}$.

2 Fourth moment calculations

We start by reporting the Laplace transform of (31) arising from three contributions in (19).
The first reads

$$\frac{4!}{2^4}\left[\sum_{\substack{n_1,n_2=-M\\ n_1\neq\pm n_2}}^{M} U_{k_{n_1}}^2 \frac{U_{k_{n_2}}^2}{s^3(s+k_{n_1}^2 D)(s+k_{n_2}^2 D)} + \sum_{n_1=-M}^{M} \frac{U_{k_{n_1}}^4}{s^3(s+k_{n_1}^2 D)^2}\right],$$

the second is

$$\frac{4!}{2^4}\left\{\sum_{\substack{n_1,n_2=-M\\ n_1\neq\pm n_2\\ n_1\neq -\frac{n_2}{2}\\ n_1\neq -2n_2}}^{M} \frac{U_{k_{n_1}}^2 U_{k_{n_2}}^2}{s^2(s+k_{n_1}^2 D)(s+k_{n_2}^2 D)[s+(k_{n_1}+k_{n_2})^2 D]} + \right.$$

$$+\sum_{n_1=-M}^{M} U_{k_{n_1}}^4 \left[\frac{1}{s^3(s+k_{n_1}^2 D)^2} + \frac{1}{s^2(s+k_{n_1}^2 D)^2(s+4k_{n_1}^2 D)}\right] +$$

$$\left. + \sum_{n_1=-M/2}^{M/2} \frac{2U_{k_{n_1}}^4}{s^2(s+k_{n_1}^2 D)^2(s+4k_{n_1}^2 D)}\right\},$$

and the third

$$\frac{4!}{2^4}\left[\sum_{\substack{n_1,n_2=-M\\ n_1\neq -n_2\\ n_1\neq -\frac{n_2}{2}}}^{M} \frac{U_{k_{n_1}}^2 U_{k_{n_2}}^2}{s^2(s+k_{n_1}^2 D)^2[s+(k_{n_1}+k_{n_2})^2 D]} + \sum_{n_1=-M}^{M} \frac{U_{k_{n_1}}^4}{s^3(s+k_{n_1}^2 D)^2} + \right.$$

$$\left. + \sum_{n_1=-M/2}^{M/2} \frac{U_{k_{n_1}}^4}{s^2(s+k_{n_1}^2 D)^3}\right].$$

In analogy with the analysis performed for the third moment, we only retain the part of the Laplace transform $\propto L^2$:

$$\frac{4!}{2^4}\sum_{n_1,n_2=-M}^{M} U_{k_{n_1}}^2 U_{k_{n_2}}^2 \left\{ \frac{1-\delta_{n_1\pm n_2}}{s^3(s+k_{n_1}^2 D)(s+k_{n_2}^2 D)} + \frac{(1-\delta_{n_1+n_2})\left(1-\delta_{n_1+\frac{n_2}{2}}\right)}{s^2(s+k_{n_1}^2 D)^2[s+(k_{n_1}+k_{n_2})^2 D]} + \right.$$

$$\left. + \frac{(1-\delta_{n_1\pm n_2})\left(1-\delta_{n_1+\frac{n_2}{2}}\right)(1-\delta_{n_1+2n_2})}{s^2(s+k_{n_1}^2 D)(s+k_{n_2}^2 D)[s+(k_{n_1}+k_{n_2})^2 D]} \right\}. \tag{S4}$$

Now we need to invert back in time the leading part of the Laplace transform (S4). Inverting the first of the three terms appearing in (S4), we have

$$\frac{4!}{2^2}\sum_{\substack{n_1,n_2=1\\ n_1\neq n_2}}^{M} U_{k_{n_1}}^2 U_{k_{n_2}}^2 \left\{ \frac{t^2}{2k_{n_1}^2 k_{n_2}^2 D^3} - \frac{t(k_{n_1}^2+k_{n_2}^2)}{k_{n_1}^4 k_{n_2}^4 D^3} + \frac{1-e^{-k_{n_1}^2 Dt}}{k_{n_1}^6(k_{n_2}^2-k_{n_1}^2)D^4} + \frac{1-e^{-k_{n_2}^2 Dt}}{k_{n_2}^6(k_{n_1}^2-k_{n_2}^2)D^4} \right\},$$

the second yields

$$\frac{4!}{2^4}\sum_{\substack{n_1,n_2=-M\\ n_1\neq\pm n_2\\ n_1\neq -n_2/2}}^{M} U_{k_{n_1}}^2 U_{k_{n_2}}^2 \left\{ \frac{t}{k_{n_1}^4 D^3}\left[\frac{1}{(k_{n_2}-k_{n_1})^2} + \frac{e^{-k_{n_1}^2 Dt}}{k_{n_2}^2+2k_{n_2}k_{n_1}}\right] - \right.$$

$$\left. (1-e^{-k_{n_1}^2 Dt})\frac{(2k_{n_2}^2+4k_{n_2}k_{n_1}-k_{n_1}^2)}{k_{n_1}^6(k_{n_2}^2+2k_{n_1}k_{n_2})D^4} - \frac{1-e^{-(k_{n_1}+k_{n_2})^2 Dt}}{(k_{n_1}+k_{n_2})^4(k_{n_2}^2+2k_{n_1}k_{n_2})^4 D^4} \right\},$$

and the last one

$$\frac{4!}{2^4}\sum_{\substack{n_1,n_2=-M\\ n_1\neq\pm n_2\\ n_1\neq -n_2/2\\ n_1\neq -2n_2}}^{M} U_{k_{n_1}}^2 U_{k_{n_2}}^2 \left\{ \frac{t}{k_{n_1}^2 k_{n_2}^2 D^3} + \frac{1-e^{-k_{n_1}^2 Dt}}{k_{n_1}^4(k_{n_1}^2-k_{n_2}^2)(k_{n_2}^2+2k_{n_1}k_{n_2})D^4} + \right.$$

$$\left. \frac{1-e^{-k_{n_2}^2 Dt}}{k_{n_2}^4(k_{n_2}^2-k_{n_1}^2)(k_{n_1}^2+2k_{n_1}k_{n_2})D^4} - \frac{1-e^{-(k_{n_1}+k_{n_2})^2 Dt}}{(k_{n_1}+k_{n_2})^4(k_{n_2}^2+2k_{n_1}k_{n_2})(k_{n_1}^2+2k_{n_1}k_{n_2})} \right\}.$$

Hence, the limiting behaviours can be easily derived. For short times ($t \ll \frac{L^2}{M^2 D}$), one has $\langle\langle[x(t)-x(0)]^4\rangle_w\rangle_\phi \sim L^2 t^4$, while for $t \gg \frac{L^2}{D}$, $\langle\langle[x(t)-x(0)]^4\rangle_w\rangle_\phi \sim L^2 t^2$. On the other side, the study of intermediate times scaling behavior requires to consider only the terms proportional to $1-e^{-k_{n_1}^2 Dt}$. Therefore, approximating the double summation with a double integral and applying the change of variables $y = Dk_1^2 t$ and $z = Dk_2^2 t$ in analogy with (S3), yields that $\langle\langle[x(t)-x(0)]^4\rangle_w\rangle_\phi \sim L^2 t^{3-\gamma}$.

The analysis of $\langle\langle[x(t)-x(0)]^4\rangle_w\rangle_0$ in the case of zero disorder must be performed in analogy with the other cases analyzed. Hence, considering the definition (33), we need to calculate the Laplace transform of the term

$$\sum_{\substack{n_1,n_2,n_3=-M \\ n_1\neq -n_2 \\ n_1\neq -n_3 \\ n_2\neq -n_3}} f(\phi_{n_1}=0,\phi_{n_2}=0,\phi_{n_3}=0,\phi_{n_1-n_2-n_3}=0;t).$$

A rather lengthy calculation yields

$$\begin{aligned}
&\frac{4!}{2^4}\left\{\sum_{\substack{n_1,n_3=-M \\ n_1\neq n_3/2}}^{M} \frac{U_{k_{n_1}}U_{k_{-2n_1}}U_{k_{n_3}}U_{k_{n_1-n_3}}}{s^2(s+k_{n_1}^2 D)^2[s+(k_{n_1}-k_{n_3})^2 D]} + 2\sum_{n_1=-M/2}^{M/2}\frac{U_{k_{n_1}}^2 U_{k_{2n_1}}^2}{s^2(s+k_{n_1}^2 D)^3} + \right. \\
&+\sum_{\substack{n_1,n_2=-M \\ n_1\neq -n_2/2}}^{M}\left[\frac{U_{k_{n_1}}^2 U_{k_{n_2}}U_{k_{-2n_1-n_2}}}{s^2(s+k_{n_1}^2 D)^2[s+(k_{n_1}+k_{n_2})^2 D]} + \frac{U_{k_{n_1}}U_{k_{n_2}}U_{k_{-2(n_1+n_2)}}U_{k_{n_1+n_2}}}{s^2(s+k_{n_1}^2 D)[s+(k_{n_1}+k_{n_2})^2 D]^2}\right] + \\
&\left. + \sum_{\substack{n_1,n_2,n_3=-M \\ n_1\neq -n_2 \\ n_1\neq -n_3 \\ n_1\neq -n_2/2 \\ n_1\neq n_3/2 \\ n_1\neq -(n_2+n_3)/2 \\ n_2\neq -n_3 \\ n_3\neq -2(n_1+n_2)}}^{M} \frac{U_{k_{n_1}}U_{k_{n_2}}U_{k_{n_3}}U_{k_{-n_1-n_2-n_3}}}{s^2(s+k_{n_1}^2 D)[s+(k_{n_1}+k_{n_2})^2 D][s+(k_{n_1}+k_{n_2}+k_{n_3})^2 D]}\right\}.
\end{aligned} \tag{S5}$$

Owing to the analysis reported above, the leading term is the last one $\propto L^3$, which also surmounts the $\langle\langle[x(t)-x(0)]^4\rangle_w\rangle_\phi$ contributions that are $\propto L^2$ (see Eq.(32)). Therefore, let us invert back in time the last summation in (S5):

$$\frac{4!}{2^4}\sum_{\substack{n_1,n_2,n_3=-M\\ n_1\neq -n_2\\ n_1\neq -n_3\\ n_1\neq -n_2/2\\ n_1\neq n_3/2\\ n_1\neq -(n_2+n_3)/2\\ n_2\neq -n_3\\ n_3\neq -2(n_1+n_2)}}^{M} U_{k_{n_1}}U_{k_{n_2}}U_{k_{n_3}}U_{k_{-n_1-n_2-n_3}}\left\{\frac{t}{k_{n_1}^2(k_{n_1}+k_{n_2})^2(k_{n_1}+k_{n_2}+k_{n_3})^2D^3}-\right.$$
$$-\frac{1-e^{-k_{n_1}^2Dt}}{k_{n_1}^4(k_{n_2}+2k_{n_1}k_{n_2})[(k_{n_2}+k_{n_3})^2+2k_{n_1}(k_{n_2}+k_{n_3})]D^4}+$$
$$+\frac{1-e^{-(k_{n_1}+k_{n_2})^2Dt}}{(k_{n_1}+k_{n_2})^4(k_{n_2}+2k_{n_1}k_{n_2})[k_{n_3}^2+2k_{n_3}(k_{n_1}+k_{n_2})]D^4}-$$
$$\left.-\frac{1-e^{-(k_{n_1}+k_{n_2}+k_{n_3})^2Dt}}{(k_{n_1}+k_{n_2}+k_{n_3})^4[(k_{n_2}+k_{n_3})^2+2k_{n_1}(k_{n_2}+k_{n_3})][k_{n_3}^2+2k_{n_3}(k_{n_1}+k_{n_2})]}.\right\}$$

In the short time, limit $t\ll\frac{L^2}{M^2D}$), we have $\langle\langle[x(t)-x(0)]^4\rangle_w\rangle_0\sim L^3t^4$. For long times ($t\gg\frac{L^2}{M^2D}$) $\langle\langle[x(t)-x(0)]^4\rangle_w\rangle_0\sim L^3t$. For times such that $\frac{L^2}{M^2D}\ll t\ll\frac{L^2}{M^2D}$ we need to approximate the sum with a triple integral and change variables according to $y=Dk_1^2t$, $z=Dk_2^2t$ and $q=Dk_3^2t$, achieving the scaling form $\langle\langle[x(t)-x(0)]^4\rangle_w\rangle_0\sim L^3t^{3-\frac{5\gamma}{4}}$.

References

[1] F. Cecconi, G. Costantini, A. Taloni, and A. Vulpiani, "Probability distribution functions of sub-and superdiffusive systems," *Physical Review Research*, vol. 4, no. 2, p. 023192, 2022.
